# Antimicrobial multidrug resistance of *Escherichia coli* from broiler farms in Zhanjiang, China

**DOI:** 10.1371/journal.pone.0335518

**Published:** 2025-11-03

**Authors:** CuiYi Liao, JinJu Peng, Shuaishuai Luo, Xingpeng Xie, Yang Li, Haotian Ma, Mengbo Yu, Yuexia Ding, Yi Ma

**Affiliations:** 1 College of Coastal Agricultural Sciences, Guangdong Ocean University, Zhanjiang, China; 2 College of Traditional Chinese Medicine, Zhanjiang University of Science and Technology, Zhanjiang, China; Universidad San Francisco de Quito, ECUADOR

## Abstract

Guangdong Province is an important area of poultry breeding in China. Zhanjiang city is located in the western part of Guangdong Province, where there are many broiler farms. To investigate antimicrobial resistance (AMR) and the presence of resistance genes in *Escherichia coli* from broiler farms, a total of 220 samples were collected from soil and feces at eight broiler farms. Subsequently, 220 strains of *E. coli* were isolated for drug resistance analysis and detection of AMR genes. The results revealed that the isolated *E. coli* strains exhibited high prevalence of multidrug resistance to 12 antimicrobial drugs including amoxicillin, tetracycline, cotrimoxazole and sulfisoxazole. Among the isolated strains, 95% of the isolates were resistant to more than three antimicrobial agents; notably, thirty-nine strains showed multidrug resistance to ten tested drugs, while four strain exhibits multidrug resistance to as many as fifteen antibacterial drugs. Additionally, seven AMR genes such as *bla*_*TEM*_ and *sul2* were detected in over half (≥50%) of the isolated *E. coli* samples; thirteen AMR genes had relatively low detection prevalence (≤30%). Correlation analysis indicated a strong association between certain AMR genes (*bla*_*TEM*_, *pexA, aadA1, bla*_*AIM*_, *ant(3")-I, sul2, sul3, tet(D)*) and AMR (≥50%). In conclusion, *E.coli* strains obtained from soil and fecal samples in broiler farms exhibited multidrug resistant phenotypes along with carrying various AMR genes. This provides a reference for the scientific control of *E. coli* multidrug resistance in this area.

## Introduction

*Escherichia coli* is a member of the *Enterobacteriaceae* family, which is widely distributed throughout the entire chicken farming feeding chain, livestock houses, feces, and surrounding soil environments. It can cause significant economic losses to the livestock industry and pose serious threats to global public health security. According to the World Health Organization (WHO) classification of the expression of antibiotics, the use of antibiotics in animals can be divided into three major categories: therapeutic use, disease prevention, and promoting economic growth [1]. In the poultry industry, antibiotics are mainly used to treat intestinal infections caused by *Salmonella* or *E. coli*. Researchers investigated the antibiotic usage and resistance issues in chicken farms in Guangdong Province, China. The primary classes of antibiotics utilized included macrolides, sulfonamides, quinolones, chloramphenicols, and tetracyclines [2]. Additionally, the researchers observed that the contamination levels of antibiotics and AMR genes were more severe in broiler farms than in layer farms [2]. Antibiotic usage for disease prevention is permitted in all large poultry-producing countries [3]. The shift to intensive livestock farming will rely heavily on antibiotics to keep animals healthy, with global antibiotic consumption expected to increase by 67% between 2010 and 2030 [4].The report shows that China was considered the biggest user of antibiotics in the world, with antibiotic use in animal was about 5.7 times that in the United States [5]. Chinese animal surveillance revealed high levels of antimicrobial resistance and increasing resistance trends in bacteria of animal origin [5].

The emergence of AMR has raised significant concerns regarding the misuse of antibiotics. The utilization of antimicrobials in livestock is closely associated with the development of antimicrobial resistance, and mechanisms for antibiotic resistance can easily disseminate within microbial communities [6]. While studies on antimicrobial resistance have primarily focused on clinical pathogens, there has been a recent surge in attention towards its impact on animals, agricultural practices, wildlife, and the environment due to the rise of zoonotic diseases [7], the development of resistant strains has raised some public health concerns. With the increasing recognition of the “One Health” concept, the environmental risks associated with the dissemination of antibiotic-resistant bacteria from livestock farming have garnered significant attention. Extensive resistome reservoirs have been identified in various farm manures, wastewater, and surrounding environments [8,9]. Antibiotic-resistant strains can be transmitted to humans via food chains, water contamination and other pathways, thereby posing a substantial threat to human health and safety. In particular, multidrug resistant *E. coli* has emerged as a critical pathogen endangering public health [10,11].

*E.coli* is facultative, anaerobic Gram-negative rods commonly found in the intestinal tract of food-production animals and humans. *E.coli* can be classified into different pathotypes capable of causing various diseases. Intestinal pathogenic *E. coli* (IPEC) is responsible for gastrointestinal disorders ranging from mild diarrhea to severe colitis [12]. Extraintestinal pathogenic *E. coli* (ExPEC) is a new group of *E. coli* that colonizes other tissues outside the host intestine and causes serious disease. Avian pathogenic *E. coli* (APEC), a subset of ExPEC, primarily causes respiratory or systemic infections in poultry [13]. Moreover, it is a major contributor to colibacillosis in poultry production, which leads to decreased productivity and increased mortality resulting in significant economic losses [14]. Colibacillosis manifests as acute fatal septicemia or sub-acute fibrinous pericarditis, airsacculitis, salpingitis, and peritonitis. Prolonged use of antibiotics not only promotes resistance among pathogenic bacteria but also induces toxic side effects on tissues and organs, thereby impacting production performance and causing substantial economic losses to the breeding industry. Furthermore, the frequent application of similar antimicrobial drugs for treating *E. coli* induced diseases in animals and humans has made it challenging to identify effective antimicrobial agents against bacterial infections in humans [15].

Zhanjiang, located in Guangdong Province, is a significant poultry producer in China. However, limited knowledge exists regarding multidrug resistance patterns and the distribution of AMR genes among *E. coli* isolates from broiler farms and their surroundings. This study aimed to determine the distribution of AMR genes and analyse multidrug resistance profiles among *E. coli* strains isolated from broiler farms, providing a basis for subsequent healthy breeding or prevention and control of bacterial drug resistance.

## Materials and methods

### Sampling

Samples were obtained from eight broiler farms situated in the counties around Zhanjiang City. Farms with a breeding scale exceeding 5,000 were selected as sampling sites. The broiler has a certain range of activities, non-scale cage in the farms. The sampling time is generally selected in the morning, and the experimenters carry sterile cotton swabs and test tubes. Collect fresh and independent fecal samples after the chickens moved around. Soil samples were collected at a depth of 1–3 cm from the surface of the surrounding farms. The samples were independently collected and the weight each sample was more than 20 g. After collection, they were placed in ice boxes and brought back to the laboratory for operation within 5 hours. The remaining experimental samples were stored at 4 °C for future use and were discarded three days post-sampling. A total of 220 samples were obtained for analysis, comprising fifteen soil and fifteen fecal samples from each of six broiler farms, while ten soil and ten fecal samples from each of the remaining two farms. The sampling procedure was approved by the College of Coastal Agricultural Sciences of Guangdong Ocean University and consent was obtained from the farms during sampling.

### Isolation, culture and purification of *E. coli*

After mixing 10 g of the sample with 1 mL of ultrapure water, single colonies were isolated on McConkey medium (Beijing Land Bridge Technology Co. LTD, CM908) using the streak plate technique, and incubated at 37 °C for 18 hours. A single bright peachy or reddish, dark peachy center, round, flat, neat edges, smooth, moist surface typical colony was selected for line purification on Eosin-Methylene Blue medium (Beijing Land Bridge Technology Co. LTD, CM105). Subsequently, colony with a black metallic lustre was selected for secondary purification on Eosin-Methylene Blue medium. Next, a suspected *E. coli* single colony was selected and inoculated into a 2 mL tube containing Nutrient Broth (Beijing Land Bridge Technology Co. LTD, CM106), followed by cultivation in a 37 °C incubator for 12 hours. Finally, 30% glycerol was added for storage at −20 °C [16].

### PCR identification of *E. coli*

According to the instructions provided by the TIANamp Bacteria DNA Kit (Tiagen Biochemical Technology (Beijing) Co., Ltd, DP302), genomic DNA from each strain was extracted as a template. The preserved strains were subsequently identified through PCR utilizing the *E. coli*-specific *phoA* gene, the primer sequences for the *phoA* gene were F: 5′-TACAGGTGACTGCGGGCTTATC-3′, R: 5′-CTTACCGGGCAATA CACTCACTA-3′ [16]. The PCR reaction conditions with reference to the relevant literature [12]. The positive control was the *E. coli* quality control strain (ATCC 25922), while nuclease-free deionized water served as the negative control.

### Drug susceptibility testing

Disk diffusion method as recommended by the CLSI (Clinical and Laboratory Standards Institute), was employed for the detection and analysis of multidrug resistance. *E. coli* control strain (ATCC 25922) was used in antimicrobial susceptibility testing. Antimicrobial agents included Amoxicillin (20 μg), Carbenicillin (100 μg), Cefradine (30 μg), Cefixime (5 μg), Meropenem (10 μg), Imipenem (10 μg), Florfenicol (30 μg), Chloramphenicol (30 μg), Amikacin (30 μg), Neomycin (30 μg), Cotrimoxazole (23.75/1.25 μg), Sulfamisoxazole (300 μg), Tetracycline (30 μg), Doxycycline (30 μg), Norfloxacin (10 μg), Enrofloxacin (10 μg), Fosfomycin (200 μg), Furazolidone (300 μg). The antibiotic disks were purchased from Hangzhou Microbiology Co., Ltd. Five antimicrobial disks were placed in each Mueller-Hinton agar (Beijing Land Bridge Technology Co. LTD, CM106, CM902) plate, and the distance between the disks was greater than 24 mm with each drug subjected to three replicates for each bacterial strain, followed by cultivation in a 37 °C incubator for 20 hours. The diameter of the inhibition zone was determined using a vernier caliper, calculated as the mean of three independent measurements. The experimental methods with reference to the relevant literature [16,17]. The results were classified according to the CLSI M100-Ed32 standard [18], the number of drug resistant strains was statistically analyzed. Strains exhibiting multidrug resistance to three or more antimicrobial agents were designated as multidrug resistant (MDR).

### AMR gene detection of *E. coli*

The PCR method was used for the detection of 22 AMR genes carried by the *E. coli*. The identified AMR genes conferred resistance to several antibiotic classes, including β-lactams (*bla*_*CTX-M*_, *bla*_*TEM*_, *bla*_*CMY*_) [19–21], carbapenems (*bla*_*BIC*_, *bla*_*AIM*_, *bla*_*GIM*_) [22,23], aminoalcohols (*fexA*, *fexB*, *catB*, *pexA*) [24,25], aminoglycosides (*rmtB*, *aadA1*, *aph (3')-IIIa*, *ant (3") -I*) [26–28], sulfonamides (*sul2*, *sul3*) [29], tetracyclines (*tet(B)*, *tet(D)*, *tet(E)*) [30], quinolones (*aac(6’)-Ib-cr*, *qepA*, *qnrB*) [31]. Primer information was shown in Table 1. The positive control was the *E. coli* quality control strain (ATCC 25922), while nuclease-free deionized water served as the negative control. Due to the scarcity of specific positive control strains for some rare resistance genes in domestic and international strain repositories, this experiment was unable to include corresponding gene-specific positive controls in the detection of these genes. Only the *E. coli* (ATCC 25922) was used for verification. This situation has certain limitations. Theoretically, it may cause a slight uncertainty in the interpretation of negative results for these genes and cannot completely rule out the potential risk of false negatives.

**Table 1:** Information of PCR primers for *E. coli* resistance genes.

Class	Gene	Primer sequence	Fragment length (bp)	Annealing temperature (°C)
β-Lactams	*bla*_*CTX-M*_	F: ATGATGAAAAAATCGTTATGC R: CAGCATCTCCCAGCCTAAT	882	60
	*bla*_*TEM*_	F: ATTTCGGTGTCGCCCTTAT R: CTACGATACGGGAGGGCTTA	759	54
	*bla*_*CMY*_	F: ATGATGAAAAAATCGTTATGCT R: CAGCATCTCCCAGCCTAAT	875	60
Carbapenems	*bla*_*BIC*_	F: TATGCAGCTCCTTTAAGGGC R: TCATTGGCGGTGCCGTACAC	537	52
	*bla*_*AIM*_	F: CTGAAGGTGTACGGAAACAC R: GTTCGGCCACCTCGAATTG	322	52
	*bla*_*GIM*_	F: TCGACACACCTTGGTCTGAA R: AACTTCCAACTTTGCCATGC	1212	57
Aminoalcohols	*fexA*	F: GTACTTGTAGGTGCAATTACGGCTGA R: CGCATCTGAGTAGGACATAGCGTC	1272	57
	*fexB*	F: TTCCCACTATTGGTGAAAGGAT R: GCAATTCCCTTTTATGGACGTT	787	55
	*catB*	F: TGAACACCTGGAACCGCAGAG R: GCCATAGTAAACACCGGAGCA	547	51
	*pexA*	F: GCAGCGTGCCTTTAACATCC R: AGAAGAAGCATACCCGTGAAC	310	59
Aminoglycosides	*rmtB*	F: AACCCCTTGGCGCTATACGAG R: CGTAGTTCGCCTCCATGCCTT	779	58
	*aadA1*	F: ACCTTTTGGAAACTTCGGCTT R: CTCGCCTTTCACGTAGTGGAC	721	66
	*aph (3')-1*	F: AATTTATGCCTCTTCCGACCA R: ACAACCTATTAATTTCCCCTCGT	413	56
	*ant (3")- I*	F: TGATTTGCTGGTTACGGTGAC R: CGCTATGTTCTCTTGCTTTTG	284	55
Sulfonamides	*sul2*	F: GCGCTCAAGGCAGATGGCATT R: GCGTTTGATACCGGCACCCGT	580	56
	*sul3*	F: AGATGTGATTGATTTGGGAGC R: TAGTTGTTTCTGGATTAGAGCCT	443	60
Tetracyclines	*tet(B)*	F: CTCAGTATTCCAAGCCTTTG R: CTAAGCACTTGTCTCCTGTT	480	56
	*tet(D)*	F: ATTACACTGCTGGACGCGAT R: CTGATCAGCAGACAGATTGC	419	60
	*tet(E)*	F: GTGATGATGGCACTGGTCAT R: CTCTGCTGTACATCGCTCTT	557	52
Quinolones	*Aac (6’) -Ib-cr*	F: AACTTTCTCTCTCTATTCTTATTT R: TTGCGATGCTCTATGAGTGGCTA	500	55
	*qepA*	F: CGGCGGCGTGTTGCTGT R: CCGACAGGCCCACGACG	417	53
	*qnrB*	F: GATCGTGAAAGCCAGAAAGG R: ACGATGCCTGGTAGTTGTCC	309	53

### Correlation detection between AMR genes and drug resistance phenotypes

The results of AMR gene detection and drug susceptibility test were compared, the coincidence rate was calculated, and the correlation between AMR gene and drug resistance phenotype was analyzed. Coincidence rate (%) = (number of resistant strains with positive genes + number of sensitive strains with negative genes)/ total number of strains × 100% [32].

### Statistical analysis

The statistical analyses data were managed using Excel 2012 software. The statistical analysis was performed using SPSS 25.0 software.

## Results

### Isolation and Identification of *E. coli*

The colonies typically appeared pink, smooth, moist, and round on MacConkey agar medium, with a diameter of approximately 1.5 mm. The colonies appear purplish black on Eosin-Methylene Blue agar medium, with metallic luster, round colonies, about 1 mm in diameter. One *E. coli* strain was isolated from each sample of soil and faeces, totaling to 220 strains. The bacterial genome was extracted, followed by PCR identification. A 622 bp band was obtained, which was consistent with the fragment size of the specific primers *phoA.* It was determined that the isolated and cultured 220 strains were *E. coli*.

### Antimicrobial drug resistance of *E. coli*

The antimicrobial drug resistance results of the isolated *E. coli* strains were presented in Tables 2 and 3. The *E. coli* strains isolated from the eight broiler farms exhibited high prevalence of MDR against aminoglycosides, sulfonamides and tetracyclines. Extensively drug resistant (XDR) was defined as non-susceptibility to at least one agent in all but two or fewer antimicrobial categories and pandrug resistant (PDR) was defined as non-susceptibility to all agents in all antimicrobial categories [33]. Among the *E. coli* isolates from fecal samples, farm V exhibited the highest resistance prevalence, with over 65% multidrug resistance to all tested antibiotics. Notably, 8 antibiotics showed a complete multidrug resistance prevalence. The isolates from Farm VIII showed XDR, a complete multidrug resistance prevalence (100%) for 6 antibiotics. Regarding the *E.coli* isolates found in soil samples, farm Ⅰ and VIII displayed the highest multidrug resistance prevalence with over 80% resistant to 10 antibiotics, among them, 6–7 antibiotics had a complete multidrug resistance prevalence (100%).

**Table 2:** Results of drug sensitivity test on isolates from fecal samples of eight broiler farms.

Drug category	Drugs	Drug resistance prevalence %							
		Farm Ⅰ	Farm Ⅱ	Farm Ⅲ	Farm Ⅳ	Farm Ⅴ	Farm Ⅵ	Farm Ⅶ	Farm Ⅷ
β-Lactams	Amoxicillin	93.33	93.33	93.33	86.67	100	100	80	90
	Carbenicillin	86.67	86.67	100	73.33	100	100	60	90
	Cefradine	46.67	60	13.33	13.33	93.33	66.67	10	40
	Cefixime	26.67	40	13.33	20	100	46.67	10	30
Carbapenems	Meropenem	0	6.67	0	0	0	0	0	0
	Imipenem	6.67	6.67	0	6.67	13.33	0	0	60
Amidols	Florfenicol	86.67	86.67	93.33	80	100	93.33	90	100
	Chloramphenicol	80	80	93.33	80	100	93.33	90	100
Aminoglycosides	Amikacin	26.67	26.67	0	0	0	6.67	0	20
	Neomycin	73.33	93.33	100	60	93.33	100	100	90
Sulfonamides	Cotrimoxazole	100	93.33	93.33	86.67	100	100	80	100
	Sulfamisoxazole	100	100	93.33	86.67	100	100	100	100
Tetracyclines	Tetracycline	93.33	100	93.33	86.67	100	93.33	90	100
	Doxycycline	100	100	86.67	46.67	100	80	80	100
Quinolones	Norfloxacin	20	46.67	0	6.67	0	0	0	30
	Enrofloxacin	26.67	33.33	6.67	6.67	86.67	46.67	0	30
Fosfomycins	Fosfomycin	0	13.33	0	0	0	0	0	0
Nitrofurans	Furazolidone	6.67	6.67	0	0	6.67	0	0	10

**Table 3:** Results of drug sensitivity test on isolates from soil samples of eight broiler farms.

Drug category	Drugs	Drug resistance prevalence %							
		Farm Ⅰ	Farm Ⅱ	Farm Ⅲ	Farm Ⅳ	Farm Ⅴ	Farm Ⅵ	Farm Ⅶ	Farm Ⅷ
β-Lactams	Amoxicillin	100	80	86.67	86.67	66.67	86.67	90	100
	Carbenicillin	93.33	80	93.33	100	73.33	80	90	100
	Cefradine	66.67	40	40	73.33	20	6.67	20	60
	Cefixime	53.33	26.67	33.33	66.67	6.67	6.67	70	60
Carbapenems	Meropenem	13.33	0	0	0	0	0	0	0
	Imipenem	20	6.67	20	20	6.67	0	0	10
Amidols	Florfenicol	100	80	93.33	80	53.33	86.67	100	90
	Chloramphenicol	100	80	80	60	60	86.67	100	100
Aminoglycosides	Amikacin	0	6.67	33.33	13.33	13.33	6.67	20	10
	Neomycin	93.33	93.33	93.33	86.67	60	53.33	90	90
Sulfonamides	Cotrimoxazole	100	86.67	93.33	100	73.33	100	100	100
	Sulfamisoxazole	100	93.33	100	100	93.33	93.33	100	100
Tetracyclines	Tetracycline	100	80	93.33	86.67	73.33	100	90	100
	Doxycycline	100	86.67	93.33	100	80	80	80	90
Quinolones	Norfloxacin	26.67	13.33	20	0	6.67	0	20	20
	Enrofloxacin	53.33	26.67	33.33	20	6.67	6.67	20	80
Fosfomycins	Fosfomycin	0	0	0	6.67	6.67	0	10	0
Nitrofurans	Furazolidone	80	40	66.67	53.33	26.67	6.67	20	20

The drug resistance profiles of the 220 *E. coli* isolates against 18 antibacterial agents were presented in Table 4. High prevalence of resistance (≥85%) were observed for amoxicillin, tetracycline, florfenicol, doxycycline, chloramphenicol, cotrimoxazole, sulfafurazole, neomycin, clarithromycin and carbenicillin among the isolates. Conversely, low prevalence of resistance (≤30%) were detected for meropenem, amikacin, norfloxacin, enrofloxacin, imipenem and furazolidone.

**Table 4:** Resistance of 220 isolated strains of *Escherichia coli* from 8 broiler farms to 18 antimicrobial drugs.

Antimicrobial drugs	Number of Resistance Isolates (fecal)	Number of Resistance Isolates (soil)	Total	Percentage, %
Meropenem	1	2	3	1.36
Amikacin	11	14	25	11.36
Amoxicillin	102	95	197	89.55
Cefradine	58	36	94	42.73
Norfloxacin	14	14	28	12.73
Enrofloxacin	34	32	66	30
Tetracycline	104	99	203	92.27
Florfenicol	90	103	193	87.73
Imipenem	11	12	23	10.45
Cefixime	41	42	83	37.73
Doxycycline	95	98	193	87.73
Chloramphenicol	98	90	188	85.45
Fosfomycin	2	3	5	2.27
Cotrimoxazole	104	103	207	94.09
Sulfisoxazole	107	107	214	97.27
Neomycin	97	90	187	85
Furazolidone	4	45	49	22.27
Carbenicillin	97	97	194	88.18

**Fig 1:**
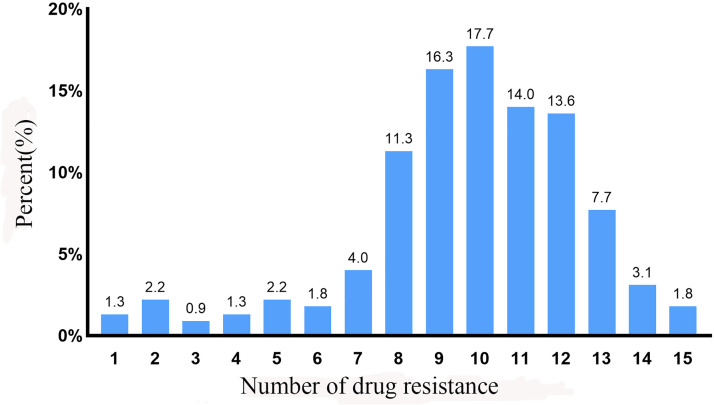
Results of multidrug resistance of 220 *Escherichia coli* isolates.

The results depicted in Fig 1 demonstrated that 95% of the isolates were resistant to more than three antimicrobial agents, displaying multidrug resistance to a maximum of 15 antibacterial agents. Moreover, over 85% of the isolated *E. coli* strains demonstrated MDR to more than eight drugs, with the highest number of strains (39 strains, accounting for 17.7%) exhibiting XDR to 10 antibacterial drugs, 14% exhibited XDR to 11 antimicrobial drugs, 13.6% demonstrated resistance to 12 drugs. Notably, only 1.8% of the strains displayed simultaneous resistance to as many as 15 drugs, while only 1.3% of the isolated were resistant to one drug.

### AMR gene distributions of *E. coli*

The detection results for the presence of 22 different AMR genes in 220 *E. coli* strains isolated from eight broiler farms are presented in Table 5. The AMR genes with a high prevalence of *E.coli* isolates in soil samples include *ant (3")- I* (72%), *sul2* (64%), *aadA1* (64%) and *bla*_*TEM*_ (61%). The high prevalence of the AMR genes of *E. coli* isolates in the fecal samples were as follows: *sul2* (89%), *bla*_*AIM*_ (77%), *ant (3")- I* (66%) and *bla*_*TEM*_ (66%). The prevalence of resistance genes associated with sulfonamides, aminoglycosides, and β-lactams was generally higher in breeding farms.

**Table 5:** Detection of drug resistance genes in 220 strains of *Escherichia coli.*

Drug category	Antimicrobial resistance genes	Number of resistance isolates			Detection prevalence %
		Fecal	Soil	Total	
β-Lactams	*bla*_*CTX-M*_	1	3	4	1.82
	*bla*_*TEM*_	72	67	139	63.18
	*bla*_*CMY*_	31	3	34	15.45
Carbapenems	*bla*_*BIC*_	2	9	11	5
	*bla*_*AIM*_	85	63	148	67.27
	*bla*_*GIM*_	0	1	1	0.45
Amidols	*fexA*	28	5	33	15
	*fexB*	0	0	0	0
	*catB*	28	19	47	21.36
	*pexA*	64	72	136	61.82
Aminoglycosides	*rmtB*	2	3	5	2.27
	*aadA1*	68	70	138	62.73
	*aph (3')-1*	6	20	26	11.82
	*ant (3")- I*	73	79	152	69.09
Sulfonamides	*sul2*	98	70	168	76.36
	*sul3*	57	62	119	54.09
Tetracyclines	*tetB*	33	20	53	24.09
	*tetD*	48	43	91	41.36
	*tetE*	14	38	52	23.64
Quinolones	*aac(6’)-Ib-cr*	3	2	5	2.27
	*qepA*	40	42	82	37.27
	*qnrB*	34	5	39	17.73

**Fig 2:**
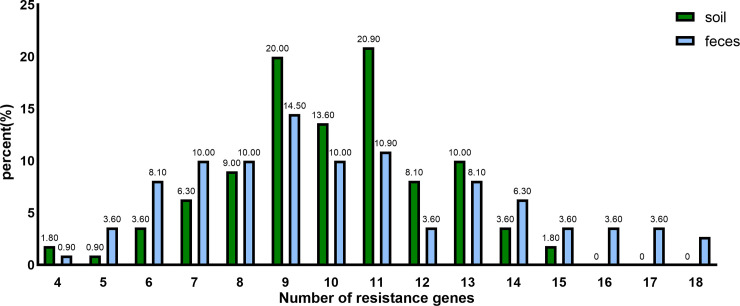
Distribution of *Escherichia coli* resistance genes in feces and soil.

The distribution of drug-resistance genes in feces and soil exhibited variations, as depicted in Fig 2, for the AMR genes carried by 220 strains of *E. coli.* Fecal isolates demonstrated a range of at least four to a maximum of eighteen different AMR genes, with the highest prevalence observed among isolated *E. coli* strains carrying nine resistance genes (16 strains, 14.5%). Soil isolates harbored at least four to a maximum of fifteen genes, with the largest proportion consisting of strains carrying 11 AMR genes (23 strains, 20.9%).

### Correlation analysis of AMR genes and drug resistance

As described in Table 6, comparative analysis of drug resistance spectrum between positive and negative strains of drug resistance genes, the following AMR genes had a high correlation rate with drug resistance phenotype: *ant (3")- I* and *sul2* were higher than 87%. The second was *bla*_*AIM*_ (71.36%). *pexA*, *aadA1*, *sul3* and *bla*_*TEM*_ were more than 60%. The correlation rate of other AMR genes was relatively low (<50%).

**Table 6:** Correlation analysis between antimicrobial resistance genes and drug resistance of *Escherichia coli* isolates.

Category	Test of antimicrobial genes		Test of multidrug resistance		Correlation rate %
	Genes	Negative(N) / positive (P)	Number of detections	Sensitive strain	
β-Lactams	*bla*_*TEM*_	P	139	4	67.27
		N	68	9	
Amidols	*pexA*	P	136	22	65.91
		N	53	9	
Aminoglycosides	*aadA1*	P	138	33	65.91
		N	42	7	
	*ant (3")- I*	P	152	16	88.18
		N	10	42	
Sulfonamides	*sul2*	P	168	3	87.73
		N	24	25	
	*sul3*	P	119	31	64.1
		N	48	22	
Tetracyclines	*tetD*	P	78	5	51.36
		N	102	35	
Carbapenems	*bla*_*AIM*_	P	146	26	71.36
		N	37	11	

## Discussion

The emergence of antibiotic resistance in *E. coli* represents a significant challenge for human and animal health on a global scale. It is a genuine public health concern that demands urgent attention [34]. The judicious use of antibiotics in humans and animal production is essential to mitigate the risk of antibiotic resistance [35]. The majority of antibiotics consumed by humans and animals are not fully metabolised within the body, resulting in their release into the environment through excretion [36]. In this study, it was observed that *E. coli* strains isolated from fecal and soil samples collected from broiler farms exhibited drug MDR and XDR. Notably, The prevalence of multidrug resistance to sulfisoxazole, cotrimoxazole, and tetracycline among the isolated strains exceeded 90%. Bratfelan et al. investigated the prevalence and antimicrobial resistance of *E. coli* isolates from chicken meat in Romania, they found that *E. coli* exhibited high resistance to tetracycline, ampicillin, and sulfamethoxazole [37]. Messaili et al. determined the antimicrobial resistance of 100 fecal *E. coli* isolated from chickens in Algeria, high resistance prevalence was noted for amoxicillin, cefazolin, fluoroquinolones, tetracycline, trimethoprim, and sulfonamides, 93% of *E. coli* isolated strains present MDR [38]. In Hebei Province, China, the highest antibiotic resistance rate was observed for ampicillin, exceeding 90.0%. Furthermore, significant resistance rates were also recorded for florfenicol, ceftiofur, enrofloxacin, and sulfisoxazole, all surpassing 70.0% [39]. Data from Zhejiang Province demonstrate that the isolated Escherichia coli strains exhibit marked resistance to tetracycline (92.92%), sulfisoxazole (93.05%), florfenicol (83.11%), and ampicillin (78.27%) [40]. These findings were similar to those of our experiment. Among these antibiotics, sulfonamides, amoxicillin and tetracyclines demonstrated the highest levels of multidrug resistance. They act as broad spectrum antibiotics, which were also commonly employed in clinical treatment of intestinal bacterial infections. There are two main ways to emergence antimicrobial resistance: the first is through intrinsic genetic mutations, mainly vertical transmission from parents to offspring, and the second is through horizontal gene transfer of mobile genetic elements, accelerating the emergence of multidrug resistant strains [41]. This may be attributed to the high multidrug resistance prevalence resulting from the frequent use of these antibiotics on farms, indicating that their clinical use should be avoided or strictly regulated. Differences in individual drug resistance appear in different regions, which may be due to factors such as the choice and dosage of drugs used on farms, feeding methods and conditions.

Ninety-five percent of the 220 isolates were resistant to more than 3 antibiotics, and a maximum of 15 antibiotics, indicating the severity of multidrug resistance. In some studies of China, high drug resistance to β-lactams including ampicillin and amoxicillin, with a resistance prevalence of more than 97%, followed by florfenicol (95%) was observed in avian-origin *E. coli* strains of Shandong Province [42]. In four provinces of Eastern China, 230 strains of avian *E. coli* were isolated and their drug resistance was detected. The resistance prevalence of the isolates to tetracycline was higher than 95% which was similar to our research results [43]. It has been observed that *E. coli* strains exhibit a high level of resistance to sulphamethoxazole-trimethoprim, followed by tetracycline and ampicillin in Bangladesh [44]. Antimicrobial multidrug resistance is closely associated with the utilization of antibiotics. Several studies have reported that *E. coli* from broiler chickens revealed multidrug resistance to ampicillin, tetracycline, ciprofloxacin, nalidixic acid, and sulfamethoxazole-trimethoprim [45]. The transmission of ARGs from bacterial organisms to potential human pathogens has significant implications for human health [46]. This is due to the fact that the presence of these genes in the environment undermines the efficacy of antibiotic treatments and consequently poses a threat to public health [47]. Experiments conducted by Chuppava et al. demonst prevalenced that broiler chicken feces harbor drug resistant *E. coli* strains carrying multiple drug resistance genes, which subsequently spread within the vicinity of chicken houses [48]. Given that *E. coli* is a prevalent pathogen in both humans and animals, the issue of AMR associated with *E. coli* has become a prominent concern in public health. The isolates obtained from eight broiler farms exhibited up to 22 AMR genes, indicating the complexity and consistency of multidrug resistance patterns. Moreover, there was a significant disparity in the detection prevalence of identical AMR genes between fecal and soil isolates from the same farm, suggesting the intricate nature of drug resistance gene existence. The main AMR genes carried by 220 isolates included *bla*_*TEM*_, *bla*_*AIM*_, *pexA*, *aadA1*, *ant(3")-I*, *sul2*,and *sul3*. The TEM family of extended-spectrum beta-lactamases (ESBLs) has long been recognized as globally prevalent. Plasmid-mediated beta-lactamases, *bla*_*TEM*_ was identified in the *E. coli* isolates at a prevalence of 63.18%. Among the 262 ESBL-positive *E. coli* isolates in central China, TEM accounted for the highest proportion at 76.72%, which aligns with the findings of this study [49]. TEM was also found to be one of the main genes mediating β-lactam antibiotic resistance in *E.coli* isolated in Hebei Province [39]. These genes are plasmid-borne and can be transferred to other bacteria, environments, and humans [50], making the latest generation cephalosporin ineffective. According to a review of the transmission of ESBL-producing bacteria through products of livestock origin, chicken is a significant source of infection [51]. Several studies in China and other countries had reported a high prevalence of *bla*_*TEM*_, ranging from 72% to 100% [52–54]. The protein encoded by the *pexA* gene is structural similarities to efflux pumps of the major facilitator superfamily. *E. coli* from broiler farm exhibited the highest prevalence for *aadA* (aminoglycosides) at 80% in Shandong Province of China [55]. Enzyme modification of aminoglycosides, increased efflux activity, decreased permeability, and modification of 30S ribosomal subunits are all mechanisms contributing to drug resistance. Among these, aminoglycoside-modifying enzymes (AMEs) are considered key contributors to the development of drug resistance, enzymatic modification of aminoglycosides enables bacteria to overcome their antimicrobial effects [56]. In this study, the prevalence for the aminoglycoside-modifying enzyme resistance genes *aadA1* and *ant (3")- I* were 62.73% and 69.09%. These findings suggest that the primary mechanism of aminoglycoside multidrug resistance in *E. coli* isolates from chicken samples in the Zhanjiang area is mediated by aminoglycoside-modifying enzymes. The sulfonamides were synthetic compounds of commonly used antibacterial drugs. Their broad-spectrum bacteriostatic activity against both Gram-positive and Gram-negative bacteria led to their widespread use in human and veterinary medicine worldwide, resulting in a significant issue of resistance [57]. The resistance genes carried by soil isolates were similar to those of fecal isolates in this study, which may be due to the fact that there were many pollution sources in contact with soil, which can be infiltrated by sewage and deposited by feed in addition to feces. This experiment focuses on the detection of rare antibiotic resistance genes in broiler and their environment, as they may rapidly become dominant drug-resistant types due to horizontal transmission. Additionally, these genes tend to accumulate in the environment, forming a “silent” reservoir of resistance genes. When environmental conditions change, these genes may be activated and expressed, subsequently entering farmed animals via pathways such as the food chain or direct contact, thereby facilitating the spread of antibiotic resistance. Furthermore, the evolution of environmental antibiotic resistance exhibits a lag effect, meaning that currently rare resistance genes could potentially emerge as significant future risk factors. By detecting these rare resistance genes, it is possible to construct a more comprehensive map of environmental antibiotic resistance, identify potential high-risk genes and transmission routes, and provide a foundation for early prevention and control strategies.

Antimicrobial resistance was closely associated with the presence of resistant genes [58]. In this experiment, *bla*_*TEM*_, *bla*_*AIM*_, *pexA*, *aadA1*, *ant(3")-I*, *sul2*, and *sul3* exhibited a high correlation (>55%) with drug resistance. *E. coli* possesses a robust capacity for accumulating antimicrobial AMR genes [34]. The development of bacterial multidrug resistance is influenced by various factors including bacterial characteristics, the dissemination of AMR genes, and drug usage [15]. The regulation of bacterial multidrug resistance phenotype is influenced by multiple AMR genes, given the abundance of known drug-resistance genes in *E. coli*. Additionally, it is possible that there are other unidentified drug resistance mechanisms for certain antibiotics. Although this study detected only a limited number of AMR genes, it is likely that the complete spectrum of drug-resistance genes carried by *E. coli* isolates was not fully represented. It is also plausible that resistant bacteria may carry a low abundance of resistance genes, which may not manifest corresponding drug resistance phenotypes [59]. Alternatively, the detected resistance genes may confer only low levels of drug resistance. Most *E. coli* strains were MDR, even if a strain possesses several resistance genes, they may not be active. In addition, the resistance genes analyzed were located on mobile genetic elements, which may allow them to transfer to other microorganisms [60]. The relatively low correlation may be ascribed to regional variations in drug usage patterns or the incomplete detection of all antimicrobial resistance genes in this trial, which limits the ability to fully elucidate the correlation between resistance genes and phenotypes. Additionally, other potential factors such as variability in gene expression, silent genes, or alternative resistance mechanisms could contribute to this observation. Further investigation in future studies is warranted to ascertain the precise underlying causes.

## Conclusion

The present study investigated the multidrug resistant *E. coli* strains isolated from soil and faeces of broiler farms in Zhanjiang, China, which harbored a significant number of AMR genes. A significant correlation was observed between specific AMR genes and drug resistance phenotypes. Therefore, to mitigate the dissemination of AMR genes and the emergence of drug-resistant bacteria, it is recommended that broiler practitioners regulate the usage of various antibacterial agents, particularly tetracyclines, sulfonamides, and amide alcohols. These findings suggest that antibiotic MDR is a significant issue in Zhanjiang farms. To prevent and control the spread of antibiotic MDR and AMR genes, it is essential for poultry farms to use antibiotics judiciously under the guidance of veterinary professionals. Furthermore, there is an urgent need to explore and develop alternative products in order to improve public health conditions.

## Supporting information

S1 Table Drug resistance. (XLSX)

S2 Table Resistance gene. (XLSX)

S3 File The data tables and statistical figures of the manuscript. (DOCX)
