# Supplementary material for: Antimicrobial multidrug resistance of Escherichia coli from broiler farms in Zhanjiang, China
Source: PLoS One. 2025 Nov 3;20(11):e0335518. doi: 10.1371/journal.pone.0335518 (PMC12582493; doi:10.1371/journal.pone.0335518)
Supplement: S3 File — (DOCX) [file pone.0335518.s003.docx]

**Table 1 Information of PCR primers for E. coli resistance genes**

| Gene | Primer sequence | Fragment length（bp） | Annealing temperature (℃) |
| --- | --- | --- | --- |
| *blaCTX-M* | F: ATGATGAAAAAATCGTTATGC  R: CAGCATCTCCCAGCCTAAT | 882 | 60 |
| *blaTEM* | F: ATTTCGGTGTCGCCCTTAT  R: CTACGATACGGGAGGGCTTA | 759 | 54 |
| *BlaCMY* | F: ATGATGAAAAAATCGTTATGCT  R: CAGCATCTCCCAGCCTAAT | 875 | 60 |
| *blaBIC* | F: TATGCAGCTCCTTTAAGGGC  R: TCATTGGCGGTGCCGTACAC | 537 | 52 |
| *blaAIM* | F: CTGAAGGTGTACGGAAACAC  R: GTTCGGCCACCTCGAATTG | 322 | 52 |
| *blaGIM* | F: TCGACACACCTTGGTCTGAA  R: AACTTCCAACTTTGCCATGC | 1212 | 57 |
| *fexA* | F: GTACTTGTAGGTGCAATTACGGCTGA  R: CGCATCTGAGTAGGACATAGCGTC | 1272 | 57 |
| *fexB* | F: TTCCCACTATTGGTGAAAGGAT  R: GCAATTCCCTTTTATGGACGTT | 787 | 55 |
| *catB* | F: TGAACACCTGGAACCGCAGAG  R: GCCATAGTAAACACCGGAGCA | 547 | 51 |
| *PexA* | F: GCAGCGTGCCTTTAACATCC  R: AGAAGAAGCATACCCGTGAAC | 310 | 59 |
| *rmtB* | F: AACCCCTTGGCGCTATACGAG  R: CGTAGTTCGCCTCCATGCCTT | 779 | 58 |
| *aadA1* | F: ACCTTTTGGAAACTTCGGCTT  R: CTCGCCTTTCACGTAGTGGAC | 721 | 66 |
| *aph (3')-1* | F: AATTTATGCCTCTTCCGACCA  R: ACAACCTATTAATTTCCCCTCGT | 413 | 56 |
| *ant (3")- I* | F: TGATTTGCTGGTTACGGTGAC  R: CGCTATGTTCTCTTGCTTTTG | 284 | 55 |
| *sul2* | F: GCGCTCAAGGCAGATGGCATT  R: GCGTTTGATACCGGCACCCGT | 580 | 56 |
| *sul3* | F: AGATGTGATTGATTTGGGAGC  R: TAGTTGTTTCTGGATTAGAGCCT | 443 | 60 |
| *tetB* | F: CTCAGTATTCCAAGCCTTTG  R: CTAAGCACTTGTCTCCTGTT | 480 | 56 |
| *tetD* | F: ATTACACTGCTGGACGCGAT  R: CTGATCAGCAGACAGATTGC | 419 | 60 |
| *tetE* | F: GTGATGATGGCACTGGTCAT  R: CTCTGCTGTACATCGCTCTT | 557 | 52 |
| *aac（6'）-Ib-cr* | F: AACTTTCTCTCTCTATTCTTATTT  R: TTGCGATGCTCTATGAGTGGCTA | 500 | 55 |
| *qepA* | F: CGGCGGCGTGTTGCTGT  R: CCGACAGGCCCACGACG | 417 | 53 |
| *qnrB* | F: GATCGTGAAAGCCAGAAAGG  R: ACGATGCCTGGTAGTTGTCC | 309 | 53 |

**Table 2 Results of drug sensitivity test on isolates from fecal samples of eight broiler farms**

| Drug  category | Drugs | Drug resistance rate % | | | | | | | |
| --- | --- | --- | --- | --- | --- | --- | --- | --- | --- |
|  |  | FarmⅠ | Farm Ⅱ | Farm Ⅲ | Farm Ⅳ | Farm Ⅴ | Farm Ⅵ | Farm Ⅶ | Farm Ⅷ |
| β-Lactams | Amoxicillin | 93.33 | 93.33 | 93.33 | 86.67 | 100 | 100 | 80 | 90 |
|  | Carbenicillin | 86.67 | 86.67 | 100 | 73.33 | 100 | 100 | 60 | 90 |
|  | Cefradine | 46.67 | 60 | 13.33 | 13.33 | 93.33 | 66.67 | 10 | 40 |
|  | Cefixime | 26.67 | 40 | 13.33 | 20 | 100 | 46.67 | 10 | 30 |
| Carbapenems | Meropenem | 0 | 6.67 | 0 | 0 | 0 | 0 | 0 | 0 |
|  | Imipenem | 6.67 | 6.67 | 0 | 6.67 | 13.33 | 0 | 0 | 60 |
| Amidols | Florfenicol | 86.67 | 86.67 | 93.33 | 80 | 100 | 93.33 | 90 | 100 |
|  | Chloramphenicol | 80 | 80 | 93.33 | 80 | 100 | 93.33 | 90 | 100 |
| Aminoglycosides | Amikacin | 26.67 | 26.67 | 0 | 0 | 0 | 6.67 | 0 | 20 |
|  | Neomycin | 73.33 | 93.33 | 100 | 60 | 93.33 | 100 | 100 | 90 |
| Sulfonamides | Cotrimoxazole | 100 | 93.33 | 93.33 | 86.67 | 100 | 100 | 80 | 100 |
|  | Sulfamisoxazole | 100 | 100 | 93.33 | 86.67 | 100 | 100 | 100 | 100 |
| Tetracyclines | Tetracycline | 93.33 | 100 | 93.33 | 86.67 | 100 | 93.33 | 90 | 100 |
|  | Doxycycline | 100 | 100 | 86.67 | 46.67 | 100 | 80 | 80 | 100 |
| Quinolones | Norfloxacin | 20 | 46.67 | 0 | 6.67 | 0 | 0 | 0 | 30 |
|  | Enrofloxacin | 26.67 | 33.33 | 6.67 | 6.67 | 86.67 | 46.67 | 0 | 30 |
| Fosfomycins | Fosfomycin | 0 | 13.33 | 0 | 0 | 0 | 0 | 0 | 0 |
| Nitrofurans | Furazolidone | 6.67 | 6.67 | 0 | 0 | 6.67 | 0 | 0 | 6.67 |

**Table 3 Results of drug sensitivity test on isolates from soil samples of eight broiler farms**

| Drug  category | Drugs | Drug resistance rate % | | | | | | | |
| --- | --- | --- | --- | --- | --- | --- | --- | --- | --- |
|  |  | FarmⅠ | Farm Ⅱ | Farm Ⅲ | Farm Ⅳ | Farm Ⅴ | Farm Ⅵ | Farm Ⅶ | Farm Ⅷ |
| β-Lactams | Amoxicillin | 100 | 80 | 86.67 | 86.67 | 66.67 | 86.67 | 90 | 100 |
|  | Carbenicillin | 93.33 | 80 | 93.33 | 100 | 73.33 | 80 | 90 | 100 |
|  | Cefradine | 66.67 | 40 | 40 | 73.33 | 20 | 6.67 | 20 | 60 |
|  | Cefixime | 53.33 | 26.67 | 33.33 | 66.67 | 6.67 | 6.67 | 70 | 60 |
| Carbapenems | Meropenem | 13.33 | 0 | 0 | 0 | 0 | 0 | 0 | 0 |
|  | Imipenem | 20 | 6.67 | 20 | 20 | 6.67 | 0 | 0 | 10 |
| Amidols | Florfenicol | 100 | 80 | 93.33 | 80 | 53.33 | 86.67 | 100 | 90 |
|  | Chloramphenicol | 100 | 80 | 80 | 60 | 60 | 86.67 | 100 | 100 |
| Aminoglycosides | Amikacin | 0 | 6.67 | 33.33 | 13.33 | 13.33 | 6.67 | 20 | 10 |
|  | Neomycin | 93.33 | 93.33 | 93.33 | 86.67 | 60 | 53.33 | 90 | 90 |
| Sulfonamides | Cotrimoxazole | 100 | 86.67 | 93.33 | 100 | 73.33 | 100 | 100 | 100 |
|  | Sulfamisoxazole | 100 | 93.33 | 100 | 100 | 93.33 | 93.33 | 100 | 100 |
| Tetracyclines | Tetracycline | 100 | 80 | 93.33 | 86.67 | 73.33 | 100 | 90 | 100 |
|  | Doxycycline | 100 | 86.67 | 93.33 | 100 | 80 | 80 | 80 | 90 |
| Quinolones | Norfloxacin | 26.67 | 13.33 | 20 | 0 | 6.67 | 0 | 20 | 20 |
|  | Enrofloxacin | 53.33 | 26.67 | 33.33 | 20 | 6.67 | 6.67 | 20 | 80 |
| Fosfomycins | Fosfomycin | 0 | 0 | 0 | 6.67 | 6.67 | 0 | 10 | 0 |
| Nitrofurans | Furazolidone | 80 | 40 | 66.67 | 53.33 | 26.67 | 6.67 | 20 | 20 |

**Table 4 Resistance of 220 isolated strains of *Escherichia coli* from 8 broiler farms to 18 antimicrobial drugs**

| Antimicrobial  drugs | Number of  resistance  isolates | Percentage, % | Antimicrobial  drugs | Number of  resistance  isolates | Percentage, % |
| --- | --- | --- | --- | --- | --- |
| Meropenem | 3 | 1.36 | Cefixime | 83 | 37.73 |
| Amikacin | 25 | 11.36 | Doxycycline | 193 | 87.73 |
| Amoxicillin | 197 | 89.55 | Chloramphenicol | 188 | 85.45 |
| Cefradine | 94 | 42.73 | Fosfomycin | 5 | 2.27 |
| Norfloxacin | 28 | 12.73 | Cotrimoxazole | 207 | 94.09 |
| Enrofloxacin | 66 | 30 | Sulfisoxazole | 214 | 97.27 |
| Tetracycline | 203 | 92.27 | Neomycin | 187 | 85 |
| Florfenicol | 193 | 87.73 | Furazolidone | 53 | 24.09 |
| Imipenem | 23 | 10.45 | Carbenicillin | 194 | 88.18 |

**Table 5 Detection of drug resistance genes in 220 strains of *Escherichia coli***

| Drug category | Antimicrobial  resistance  genes | Number of resistance isolates | | | Detection rate % |
| --- | --- | --- | --- | --- | --- |
|  |  | Fecal | Soil | Total |  |
| β-Lactams | *bla_CTX-M_* | 1 | 3 | 4 | 1.82 |
|  | *bla_TEM_* | 72 | 67 | 139 | 63.18 |
|  | *bla_CMY_* | 31 | 3 | 34 | 15.45 |
| Carbapenems | *bla_BIC_* | 2 | 9 | 11 | 5 |
|  | *bla_AIM_* | 85 | 63 | 148 | 67.27 |
|  | *bla_GIM_* | 0 | 1 | 1 | 0.45 |
| Amidols | *fexA* | 28 | 5 | 33 | 15 |
|  | *fexB* | 0 | 0 | 0 | 0 |
|  | *catB* | 28 | 19 | 47 | 21.36 |
|  | *pexA* | 64 | 72 | 136 | 61.82 |
| Aminoglycosides | *rmtB* | 2 | 3 | 5 | 2.27 |
|  | *aadA1* | 68 | 70 | 138 | 62.73 |
|  | *aph (3')-1* | 6 | 20 | 26 | 11.82 |
|  | *ant (3")- I* | 73 | 79 | 152 | 69.09 |
| Sulfonamides | *sul2* | 98 | 70 | 168 | 76.36 |
|  | *sul3* | 57 | 62 | 119 | 54.09 |
| Tetracyclines | *tetB* | 33 | 20 | 53 | 24.09 |
|  | *tetD* | 48 | 43 | 91 | 41.36 |
|  | *tetE* | 14 | 38 | 52 | 23.64 |
| Quinolones | *aac(6')-Ib-cr* | 3 | 2 | 5 | 2.27 |
|  | *qepA* | 40 | 42 | 82 | 37.27 |
|  | *qnrB* | 34 | 5 | 39 | 17.73 |

**Table 6 Correlation analysis between antimicrobial resistance genes and drug resistance of *Escherichia coli* isolates**

| Category | Test of antimicrobial resistance genes | | Test of drug resistance | | Correlation rate % |
| --- | --- | --- | --- | --- | --- |
|  | Genes | Negative(N)  / positive (P) | Number of  detections | Sensitive strain |  |
| β-Lactams | *bla_TEM_* | P | 139 | 4 | 67.27 |
|  |  | N | 68 | 9 |  |
| Amidols | *pexA* | P | 136 | 22 | 65.91 |
|  |  | N | 53 | 9 |  |
| Aminoglycosides | *aadA1* | P | 138 | 33 | 65.91 |
|  |  | N | 42 | 7 |  |
|  | *ant (3")- I* | P | 152 | 16 | 88.18 |
|  |  | N | 10 | 42 |  |
| Sulfonamides | *sul2* | P | 168 | 3 | 87.73 |
|  |  | N | 24 | 25 |  |
|  | *sul3* | P | 119 | 31 | 64.1 |
|  |  | N | 48 | 22 |  |
| Tetracyclines | *tetD* | P | 78 | 5 | 51.36 |
|  |  | N | 102 | 35 |  |
| Carbapenems | *bla_GIM_* | P | 146 | 26 | 71.36 |
|  |  | N | 37 | 11 |  |


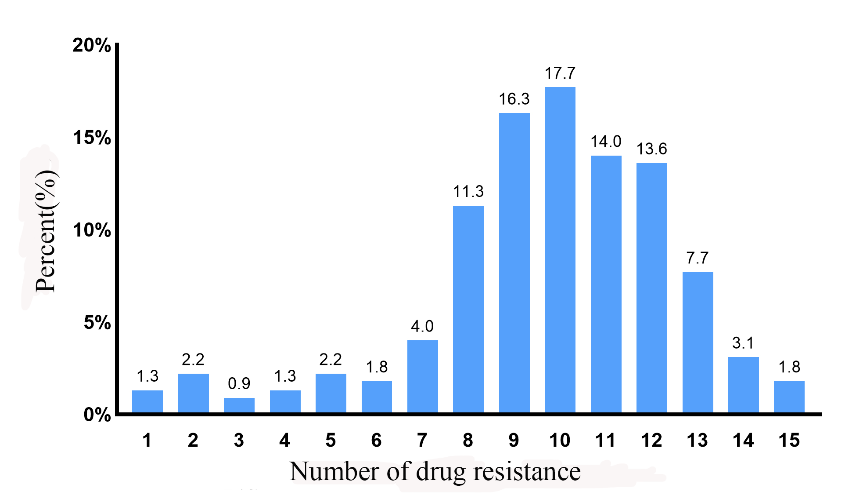


Figure 1 Results of multiple drug resistance of 220 *Escherichia coli* isolates


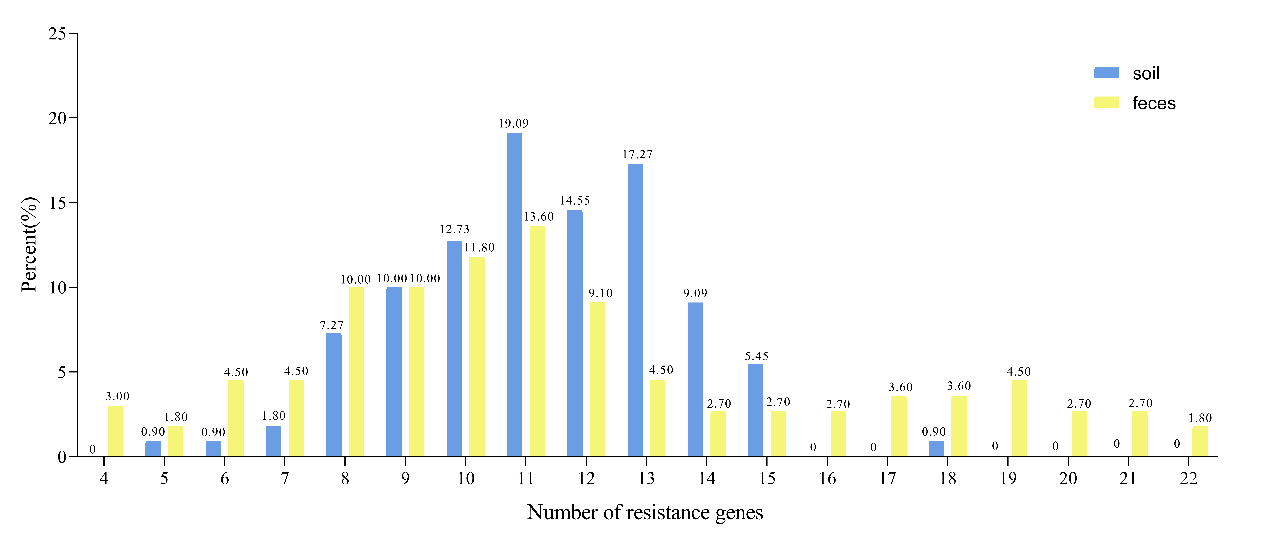


Figure 2 Distribution of *Escherichia coli* resistance genes in feces and soil
